# 

**Published:** 1873-11

**Authors:** 


### Contents of November Number.

### ORIGINAL DEPARTMENT.

ART. I.—Abstract of the Proceedings of the Buffalo Medical Association. 121
ART. II.—Clinical Remarks upon Surgical Cases in the Buffalo Hospital of the Sisters of Charity. By Prof. J. F. Miner. Reported by E. N. Brush ........ 127

### CORRESPONDENCE.

Action of Monroe County Medical Society, regarding Publication of State Society Transactions........ 131
Procidentia Uteri........ 132

### MISCELLANEOUS.

Four Cases of Albuminuria Complicating Pregnancy........ 135
Observation upon the Treatment of Yellow Fever........ 143
Remarks on Cancer of the Breast........ 149

### EDITORIAL DEPARTMENT.

The Publication of the State Medical Society Transactions........ 155
Introductory Lecture........ 155
Appointment........ 155
Election of Officers........ 155
New Operation for Aneurism........ 155

BOOKS REVIEWED:

A Manual of Medical Jurisprudence. By Alfred Swaine Taylor, M. D. Edited by John J. Reese, M. D........ 156
Chemistry, Inorganic and Organic; with Experiments. By Charles L. Bloxam........ 156
Handbook of Physiology. By William Senhouse Kirkes, M. D. Edited by W. M. Baker, F. R. S. E........ 157
Report of Columbia Hospital for Women........ 157
Pharmaceutical Lexicon. By H. V. Sweringen........ 158
Mineral Springs of the United States and Canada. By Geo. E. Walton, M. D........ 158
Clinical Electro-Therapeutics, Medical and Surgical. By Allen McLane Hamilton, M. D........ 159

PERIODICAL LITERATURE........ 159
Books and Pamphlets Received........ 160

THE BUFFALO

